# Perceived connections between information and communication technology use and mental symptoms among young adults - a qualitative study

**DOI:** 10.1186/1471-2458-10-66

**Published:** 2010-02-12

**Authors:** Sara Thomée, Lotta Dellve, Annika Härenstam, Mats Hagberg

**Affiliations:** 1 Occupational and Environmental Medicine, Sahlgrenska School of Public Health and Community Medicine, University of Gothenburg, Gothenburg, Sweden; 2 Department of Work Science, University of Gothenburg, Gothenburg, Sweden

## Abstract

**Background:**

Prospective associations have been found between high use of information and communication technology (ICT) and reported mental symptoms among young adult university students, but the causal mechanisms are unclear. Our aim was to explore possible explanations for associations between high ICT use and symptoms of depression, sleep disorders, and stress among young adults in order to propose a model of possible pathways to mental health effects that can be tested epidemiologically.

**Methods:**

We conducted a qualitative interview study with 16 women and 16 men (21-28 years), recruited from a cohort of university students on the basis of reporting high computer (n = 28) or mobile phone (n = 20) use at baseline and reporting mental symptoms at the one-year follow-up. Semi-structured interviews were performed, with open-ended questions about possible connections between the use of computers and mobile phones, and stress, depression, and sleep disturbances. The interview data were analyzed with qualitative content analysis and summarized in a model.

**Results:**

Central factors appearing to explain high quantitative ICT use were personal dependency, and demands for achievement and availability originating from the domains of work, study, social life, and individual aspirations. Consequences included mental overload, neglect of other activities and personal needs, time pressure, role conflicts, guilt feelings, social isolation, physical symptoms, worry about electromagnetic radiation, and economic problems. Qualitative aspects (destructive communication and information) were also reported, with consequences including vulnerability, misunderstandings, altered values, and feelings of inadequacy. User problems were a source of frustration. Altered ICT use as an effect of mental symptoms was reported, as well as possible positive effects of ICT on mental health.

**Conclusions:**

The concepts and ideas of the young adults with high ICT use and mental symptoms generated a model of possible paths for associations between ICT exposure and mental symptoms. Demands for achievement and availability as well as personal dependency were major causes of high ICT exposure but also direct sources of stress and mental symptoms. The proposed model shows that factors in different domains may have an impact and should be considered in epidemiological and intervention studies.

## Background

Mental health problems including anxiety and sleep disturbances have increased among the general Swedish population over the past few decades, with the highest increase seen in adolescents and young adults [1,2]. Mental disorders account for a large proportion of the disease burden in young people in all societies, and are a global public health challenge [3]. The causes of mental disorders are multi-factorial, and cultural factors seem to have an influence. One of the major changes in the exposure profiles of young people is the use of information and communication technology (ICT). In 2007, 75% of 16- to 24-year-old Swedes used the internet every day, and more than 90% had a computer at home [4]. Almost all had a mobile phone. In a qualitative study, Gustafsson et al [5] explored young adults' experience, attitudes, and health beliefs in relation to information technology use and found that they perceived both opportunities and risks, including risks to physical and psychosocial health. Positive health aspects were also described. In a prospective cohort study of young adult college and university students, participants displaying high ICT use at baseline had a higher prevalence of reported mental symptoms at the one-year follow-up, in comparison to those with low use [6]. For women, a high combined use of computer and mobile phone at baseline was associated with an increased risk of reporting symptoms of depression and prolonged stress; online chatting was associated with prolonged stress; emailing and chatting were associated with symptoms of depression; and internet surfing was associated with the prevalence of sleep disturbances. For men, the number of mobile phone calls Short Message Service (SMS) text messages sent or received per day were associated with sleep disturbances, and high SMS use was also associated with symptoms of depression at follow-up. The study concluded that ICT use may have an impact on mental health but that the causal mechanisms are unclear [6]. Earlier research has addressed the question of mechanisms. For example, it has been suggested that among some people, computer work can lead to psychophysiological stress reactions ("techno-stress") due to occupational strain, and that these reactions can become conditioned to the computer work environment leading to symptoms associated with the computer [7]. Other researchers have discussed psychological factors, such as the role of the internet in relation to mental health [8,9], and psychiatric features, such as addiction to the internet [10]. Considering the wide use of ICT, there is an urgent need to develop a model for possible explanations for the association between ICT use among young adults and symptoms of depression, sleep disturbances, and stress. A first step is to ask the young adults which connections they themselves perceive between ICT use and mental symptoms.

Our overall aim was to explore possible explanations for associations between high ICT use and symptoms of depression, sleep disorders, and stress among young adults in order to propose a model of possible pathways to mental health effects that can be tested epidemiologically. The specific purpose of this study was to explore high ICT users' own ideas and perceptions of associations between computer and mobile phone use, respectively, and stress, depression, and sleep disorders.

## Methods

This explorative qualitative study was based on semi-structured interviews with 32 young adults who in a cohort survey had reported high ICT exposure prior to reporting mental symptoms. The study was approved by the regional ethics committee for the University of Gothenburg. All participants gave written consent.

### Study group

Sixteen women and 16 men were recruited from a cohort of young adults (20-28 years) who had responded to an annual questionnaire concerning ICT exposure, demographics, and psychosocial, physical, and mental health factors, in 2004 and 2005 (n = 1843). The cohort participants were recruited from student records at colleges and universities in the southwestern and southern regions of Sweden (IT-related, medical, or nursing studies).

To be included in the present study, participants must live in the southwestern region of Sweden, have reported high computer or mobile phone exposure in 2004, and have confirmed at least two of the following mental symptoms in 2005: prolonged stress, depressive symptoms, and sleep disturbances. High ICT exposure was defined as the highest ranking reports (for men and women separately) of estimated total duration of computer use during the past week, number of mobile phone calls and text messages per day in the past week, or both. Participants must also have reported that this exposure was representative of their typical use. Mental symptom items are shown in Table 1. To enhance the potential for identifying factors or conditions connecting ICT use with mental symptoms, ten subjects were strategically included because they had reported a perceived connection between mental symptoms and IT use in response to a direct question in the cohort questionnaire. Participants were recruited consecutively until 32 individuals had been enlisted. Potential participants received a postal letter of invitation with information about the study. A week later they were telephoned and asked to participate. Monetary compensation was offered to make up for the loss of time or salary. A total of 44 individuals were contacted. Of these, eleven declined to participate: six due to lack of time, three due to current travels abroad, and two for no specified reason. One person agreed to participate but defaulted without further contact.

**Table 1:** Study demographics

	All		High computer use^1^		High mobile phone use^1^	
	n = 32		n = 28		n = 20	
	Freq	%	Freq	%	Freq	%
Gender						
*Women*	16	50	13	46	12	60
*Men*	16	50	15	54	8	40
Study background						
*IT*	25	78	25	89	13	65
*Medical doctor or nurse*	7	22	3	11	7	35
Upper quartile regular exposure in 2004						
*Computer^2^*	21	66	21	75	10	50
*Mobile phone^3^*	14	44	10	36	14	70
Reported mental symptoms in 2005^4^						
*Three symptoms*	28	88	24	86	17	85
*Two symptoms*	4	12	4	14	3	15
Psychiatric diagnosis in present study	12	38				
*Mild - moderate depression*	6	19				
*Anxiety disorder UNS*	4	12				
*Mixed anxiety and depression*	1	3				
*Mood syndrome UNS*	1	3				
Reported connection between subjective mental symptoms and IT use in 2005	10	31	10	36	6	30

freq = frequency

^1^Inclusion criteria for the groups were a reported minimum exposure in the upper half of the larger cohort in 2004 of the technology in question. Lowest exposure in High computer use group was for women 10 and for men 26 hours per week. Lowest exposure in High mobile phone use group was for women 8 and for men 7 calls or SMS messages per day. Sixteen subjects participated in both groups.

^2^For women: > 20 hours per week. For men: > 40 hours per week.

^3^For women: > 11 calls or SMS messages per day. For men: > 12 calls or SMS messages per day.

^4^Mental symptom items were: Have you continuously, for more than 7 days, during the past 12 months experienced: 1) stress (defined as a situation in which a person feels tense, restless, nervous or anxious, or is unable to sleep at night because his/her mind is troubled)? (adapted from the QPS Nordic [63]) 2a) little interest or pleasure in doing things, or 2b) feeling down, depressed, or hopeless? (adapted from Prime-MD [64]) 3a) difficulties falling asleep, or 3b) repeated awakenings with difficulties going back to sleep (adapted from the Karolinska Sleep Questionnaire [65])? All items had been adapted to reflect a prolonged state.

The high ICT users formed two subgroups: high computer users (n = 28) and high mobile phone users (n = 20). Exposure in the upper half for the technology in question was considered sufficient to qualify for each subgroup. Sixteen participants qualified for both groups. See Table 1 for study group demographics.

The high computer use group comprised 28 participants (15 men and 13 women, aged 21-28). Of these, 21 had reported regular weekly computer use in the upper quartile of the total cohort. Twenty-four had reported all three mental symptom items, and the remaining four had reported two of the mental symptoms. All 15 men and 10 of the women had a background in computer-related study; two of the other women were nurses and one was a medical doctor. Ten participants (six men and four women) had reported a perceived connection between IT use and subjective mental symptoms in the cohort questionnaire.

The high mobile phone use group comprised 20 participants (8 men and 12 women, aged 22-28). Of these, 14 had reported regular daily mobile phone use in the upper quartile of the total cohort. Seventeen had reported all three mental symptom items, and the remaining three had reported two of the mental symptoms. Seven men and six women had a background in computer-related study, one man and four women were medical doctors or students, and two women were nurses.

### Data collection

Individual semi-structured interviews were performed by the main author. The interviews took place from October 2005 to April 2006 at a university hospital clinic. The participants were asked open-ended questions about possible connections between the use of computers and mobile phones, and stress, depression, and sleep disturbances, for example: "Do you think there is a connection between the use of computers and stress? If so, how? Have you experienced that yourself? What about people in general?" In addition, direct questions were asked about the participants' own worries about personal ICT use, their experiences of problematic or destructive ICT use, and the impact of ICT use on their sleep. The interviews lasted between 40 and 90 minutes and were tape-recorded.

In parallel with and blinded to the explorative interviews, a physician performed psychiatric assessments of the participants. Psychiatric disorders were diagnosed in six men and six women [Edlund, M, unpublished data]. Mild depression was diagnosed in four participants, moderate depression in two, anxiety disorder in four, mixed anxiety in one, and unspecified mood syndrome in one participant. None of these diagnoses had previously been identified.

### Analysis

All interviews were transcribed verbatim to enable qualitative content analysis [11-13]. The interview texts were sorted at topic level to form thematic domains and entered into tables, mainly following the interview structure. Only data from high users of the ICT technology in question (computer or mobile phone) were used in the analysis of each domain. A stepwise conventional qualitative content analysis [11,12] of the data was performed by the main author (also the interviewer). The text was first condensed and coded; codes were then sorted and categories formed based on the factors and conditions described by the respondents in each domain. The process focused on a low level of interpretation of the manifest content, closely following the dictums of the subjects [13]. In the next step, the categories were compared and sorted across thematic domains to generate overall categories. Patterns and relations among the categories were sought in an iterative process, using the original interview texts as additional verification. Finally, a model was developed to illustrate and describe the categories and their relations, in order to present central features connecting ICT and mental symptoms while accounting for just about all factors mentioned by the subjects [13]. The model was developed in conjunction with the co-authors.

## Results

We first present results from the content analysis of each domain, followed by a comparison of the results between domains. Finally, we suggest a model of proposed pathways to illuminate the connections between computer and mobile phone use and mental symptoms, as perceived by the young adults.

### Computer use and stress

Most of the high computer users expressed ideas about links between computer use and stress, though some stated that these only applied to other people, and not themselves. High quantitative use, that is, long periods spent sitting at the computer, was a central link between use and symptoms. It was common for participants to spend more time than planned at the computer, either because tasks took longer than expected (leading to time pressure, tight deadlines, and overtime work), or because they became involved in internet surfing or game playing. Perceived demands and expectations, and their own desires, to be available via chat or email were also a source of high quantitative use and spending more time than intended at the computer. It was considered easy to lose perception of time and to neglect bodily signals of needs such as breaks, food, drink, or physical activity, while sitting at the computer. Furthermore, time spent at the computer was seen as time taken from other activities:

Particularly, in front of the computer you can lose perception of time. You don't feel hunger, you don't feel tired. And then if you sit in front of the computer and lose track of time and look at the clock - gosh! Well, then you end up getting stressed. Also, you don't get any energy or nutrition. You just sit there and your fingers are ice cold and you have no warmth in your feet. So in that way it could very well be harmful... and stressful... and above all, you are tired when you eventually quit. (Man A, 24 years)

Overload in communication was another problem. Chat or email messages interrupted other computer tasks, and it could be difficult to filter important messages from unimportant ones. It was difficult for participants to find time to answer all their messages as quickly as they felt expected to, and not doing so often resulted in feelings of guilt, resentment, and stress:

If someone sends an email and you haven't answered within an hour, you'll get "Did you read my email or what?" [...] You can't concentrate on anything because you get interrupted all the time. And also, you are expected to be available somehow and that can be stressful. (Woman A, 27 years)

Another stressor was managing several communications simultaneously:

You have your place in reality, where you're physically at, but then you often also have maybe four other places in the virtual world [...] where you are represented and where you need to keep up with your friends there [...] And you have to keep up with all those worlds and keep them alive and in your memory. (Woman B, 26 years)

Stress was also implied by dependency and addiction issues, such as compulsively checking messages or information, excessive game playing, online poker addiction, and feeling stressed when not connected: "It's like I'm so incredibly used to the computer. I get kind of edgy when I'm not at a computer. I feel almost naked." (Man B, 24 years)

High demands for work speed, perfection, efficiency, and being up to date, were experienced in connection with computer use. Computer use was also considered to be part of increased demands in general. Physical aspects of computer use, such as lighting and monitor displays, could also have an impact on stress (electromagnetic radiation was mentioned as a possible stressor), as could some cognitive aspects, such as time limits in computer games or the pressure to process a great deal of data in a short time. User problems concerning software or hardware, including frustrations due to slow performance or hard disk crashes, as well as competence issues such as having to learn new software, were considerable sources of stress, and also increased the time spent at the computer.

Several participants made the point that the source of stress was not computer use in itself, but rather task-, work-, or study-related demands connected with computer use, or computer use as part of meeting increased demands in general. Stress concerning the future in connection with computer use, in particular future job opportunities in IT, was also mentioned.

Along with all of the ways in which it was seen to add to stress levels, computer use was also considered to decrease stress, for example by allowing more work to be done in a shorter time.

### Computer use and depression

A central concern in discussions of computer use related to depression was high quantitative use, especially the idea of getting stuck in unproductive activities, such as game playing, which led to the feeling of having wasted time.

Well, it's kind of sad that you spend so many hours at the computer, as such. That's how I can feel after I've turned the computer off: What did I do tonight? "Yeah, I sat three hours - after spending eight hours at work!" I could have gone out and had coffee with a friend instead. That's what I feel. (Woman C, 27 years)

Demands for and expectations of availability were considered to result in communication overload, and feelings of guilt due to not being able to manage all the communication. Social isolation was another concern in relation to high computer use. A negative loop was suggested, in that already-lonely people may have a preference for using computers, which in turn could increase their tendency to lack real-life contacts and relationships, and lead to even heavier computer use. Some participants identified themselves as being in this category.

It's probably more that you ignore the other life. And that you sit at home too much and don't meet any people [...] It feels safer to sit in front of the computer for me since I find it easier to write than to communicate in real life and so I'll sit in front of the computer rather a lot. (Man C, 27 years)

High computer use was also considered to have a negative impact on physical health, leading to physical symptoms such as musculoskeletal pain, headaches, and tiredness, which in turn could increase depressive feelings. Dependency or addiction issues (e.g. online gambling) were also considered risk factors for depression, as was stress on relationships caused by one partner's heavy computer use.

As well as high quantitative use, bad quality or content of use, for example destructive information and communications, was perceived as a link between computer use and depression. This included not only bad or harmful information or misunderstandings in chat communications, but also feelings of inadequacy in the face of the unlimited possibilities and high achievements of others portrayed on the internet:

Well, if you surf the internet a lot, then you can find lots of things that can make you feel like a failure because you see how successful everybody else is, or how good looking everybody is, or how much is happening there and you can't go there. (Woman D, 27 years)

User problems and the possibility of failing to meet excessive expectations of user competence were mentioned as factors for depression. On the other hand, computer use was also considered to decrease depression through allowing easy access to social support even during times of depression and by supplying fun and entertainment. Furthermore, depressive feelings could lead to altered computer use, such as more chatting, because it could seem easier to communicate via computer than in person, or decreased chatting because of the wish not to communicate. More time could also be spent on amusements or procrastination when one was feeling down.

Stress or sleep disorders, in relation to computer use and connected to study- or work-related demands, were considered possible links from computer use to depression. There were also participants who perceived no association at all between computer use and depressive symptoms. Some pointed out that depression must depend on individual factors.

### Computer use and sleep disorders

Many participants could relate to having insufficient or dislocated sleep after sitting up late in front of the computer because of getting stuck in tasks, meeting deadlines, chatting, or game playing. It was sometimes compelling to keep up with other time zones for chatting or gambling. Several experienced difficulties relaxing after intense computer use, which in turn gave rise to difficulties falling asleep because of being too aroused or bringing task-related problems to bed. Some described seeing pictures of their recent computer activities (e.g. poker cards) when trying to go to sleep.

Because if you sit - and I've made that mistake many nights that I sit up late and chat. And then you get stuck in it - you talk to people and it's fun, and then you sit and surf around a little bit and it takes extra time, and then it's two o'clock - and you have to get up at seven. (Woman B, 26 years)

And when working on this project, for example, there were intense deadlines all the time and I ended up staying awake two days in a row and then maybe I would sleep for 24 hours after that. Or at other times I couldn't go to sleep at all, still so wound up that it would take forever to fall asleep. (Man D, 26 years)

The participants also reported that sleep could be disturbed by the compulsion to get up at night to check for messages or information (addictive or dependency factors) and by upsetting messages (destructive quality issues).

The physical impacts of computer use on sleep included noise from the computer, although most subjects turned off the computer at night or slept in another room. Less avoidable examples of physical impact were symptoms such as muscular pain or headaches related to computer use, as well as a general lack of physical activity, all of which could have negative effects on sleep. There were also comments about radiation from the computer as a possible cause of sleep disorders. Some pointed out that it was not the computer use in itself that affected sleep but task-, work-, or study-related stress, which co-varied with high quantitative use. Sleep disorders could also lead to altered computer use, for example using the computer at night when unable to sleep. It was pointed out that sleep disorders are probably dependant on individual characteristics.

### Subjective worry, possibly destructive personal use, and other effects of computer use

When asked about their own worries about personal computer use and experiences of problematic or destructive use, the participants mentioned high quantitative use resulting in wasted time, increased passivity, lost sleep, ergonomic problems, physical symptoms and health effects, high costs, addiction (e.g. compulsive chatting, game playing, information seeking or gambling), or illegal activities. However, most of those who gambled considered themselves to be in control of the problem.

Other possible mental effects of computer use mentioned were altered values because of aggressive or role-playing computer games, and vulnerability to bullying or to exploitation of their accessible personal information. Positive effects of computer use included stimulation, usefulness, entertainment, and facilitated communication.

### Summary of computer use and mental symptoms

High quantitative use was a central link between computer use and the mental symptoms described by the young adults. It was easy to spend more time than planned for at the computer (e.g. working, gaming, or chatting), and this tended to lead to time pressure, neglect of other activities and personal needs, exposure to bad ergonomics, and mental overload. The main causes of high quantitative use were personal dependency and perceived demands for and expectations of achievement and availability that originated from several domains: from work or study, from the social network or broader society, or from the participants themselves. Besides the quantitative aspects of computer use, some qualitative aspects of use were perceived as important links to mental symptoms. These included destructive information or communication, as well as online gambling and user problems that led to feelings of frustration or inadequacy. Reversed pathways between computer use and mental symptoms were also perceived, in that mental symptoms could lead to altered computer use. Computer use was also considered in some respects to actually protect against or decrease mental symptoms.

### Mobile phone use and stress

The dominant concept in interviews regarding mobile phone use was the perception by participants of demands and expectations that they be available everywhere and at all times. This could lead to high quantitative use, including interruptions of work, sleep, and other activities, the annoyance of disturbing ring signals, the feeling of never being free, and difficulties separating work from leisure:

Sometimes you want to be alone, but you can't turn it off. I never leave the apartment without the phone. It's stressful. You can't even take a normal walk to clear your thoughts and relax without the phone and somebody calling. (Woman E, 26 years)

Some experienced having so many phone calls and SMS messages that they could not make time to answer them all, which led to stress or even feelings of guilt. Participants felt that they were generally expected to explain whenever they were unavailable to answer a call or message.

You're expected to answer. And I find that pretty stressful. It rings and you can't answer, because you don't have time or you are busy. And so many people expect you to call back right away. But either you don't feel up to it or you don't have time to do it. You are so much easier to reach when you have a mobile phone. People always expect you to answer, and then when you do answer it's just, "What are you up to? Where are you? Why haven't you answered the phone? I've tried calling you lots of times!" And then you're supposed to feel guilty for not always being accessible. I find that pretty stressful. (Woman A, 27 years)

Furthermore, the flexibility of mobile phones could imply fragmentation - being overbooked and not being able to postpone. Feelings of dependency or compulsiveness in relation to the mobile phone were described, including the compulsion to check the display and feelings of high stress when not reachable (forgetting the phone at home, for example, or running out of battery power or calling range). Physical reactions, such as headaches or heat sensations after prolonged use, were also stressful and led to worries about possible hazards of electromagnetic radiation. Bad qualitative use (destructive communication or information received via the mobile phone) could also be a stressor. Relationship stress was also mentioned, in terms of the possibility of one partner keeping secrets through communications on their private phone, leading to jealousy in the other.

User competence problems mentioned as stressful included handling all the functions of the phone or trying to keep up with the latest models. Other issues were costs, worry about losing the phone, and keeping the battery charged.

All but one of the respondents had ideas about associations between mobile phone use and stress, although several could only identify those associations concerning other persons. The mobile phone was considered not only to increase, but also to decrease stress because of the flexibility it provides. Some insisted that *not *having access was the main stressor.

### Mobile phone use and depression

Respondents were less inclined to link mobile phone use to depression than to stress. A possible pathway to depression was considered to be via stress and sleep disorders, and it was suggested that the demand for constant availability could interfere with recovery. Unreturned calls or SMS messages could lead to feelings of guilt. There was also a notion that not being available could lead to being left out. Social isolation was more evident when the phone didn't ring. It was also suggested that mobile phone use might decrease personal contact "irl" (in real life) and thus increase social isolation. The quality of information or communication received via mobile phone could be destructive; for example, negative information received at the wrong time and place, or harassing phone calls or messages. It was considered easier to send negative messages (breaking up a relationship, for example) via SMS than to deliver them face to face, and so there was a perception of a higher risk of receiving bad information via mobile phone than receiving it through other channels. Jealousy in relation to others' phone use was also mentioned, as were feelings of inadequacy in response to receiving messages about others' good fortune. Electromagnetic radiation was suggested as another link to depression.

Depression or feeling down was seen as possibly leading to altered or higher use through reaching out to talk to others for support. In this sense, the mobile phone was considered to decrease depression because of the ease of reaching someone to talk to; also, it was mentioned that receiving phone calls "makes you happy".

### Mobile phone use and sleep disorders

The feeling that one was required to be constantly available was a central link between mobile phone use and sleep disturbances. A high quantity of phone calls and messages in general was perceived to lead to difficulties relaxing, and therefore to too little sleep or decreased quality of sleep. A high number of messages that had not been replied to could also lead to overload and feelings of guilt. Long phone calls before bedtime could influence sleep negatively, because of headaches or tension. However, the most obvious aspect connecting mobile phone use with sleep disturbances was being awakened at night by phone calls or messages. This meant waking up, checking the phone, and most likely answering or replying to a message. Even with the sound switched off, one could be woken by the sound of a vibrating phone or even by a blinking light. Some felt obliged to check the phone if they woke up at night, even if they had had no indication that this would be necessary. Because a mobile phone usually has only one user, some felt more obliged to answer:

If it is blinking and I see it, if I don't have my back to it, then I wake up. And, yes, I want to see who it is [...] But sometimes at night, if I wake up (and I wake up many times) then I get up and walk over to the phone to see if anybody has called or sent an SMS [laughs]. (Woman E, 26 years)

The mobile phone is personal. You know you're reaching a specific person [...] Then you are more pressured to answer, because you know it's for you, not just your house. (Woman F, 22 years)

The participants thought that people would be more likely to make late-night calls and send late-night messages to a mobile phone, in comparison to a regular phone. One opinion expressed was that leaving the mobile phone on is voluntary and implies availability: "People think that if you call a landline there is a certain time, like after ten, you can't call. With the mobile phone they think that if someone answers, then the phone is on and the person is apparently available." (Woman G, 27 years)

Other factors that were seen as possible contributors to sleep disturbance were communications with disturbing content, the thoughts provoked by a call or SMS, electromagnetic radiation, and worry about such radiation.

### Subjective worry, possibly destructive personal use, and other effects of mobile phone use

There did not seem to be a general worry about personal mobile phone use among the respondents. Some reflected upon possible health effects of electromagnetic radiation. Personal dependency on the mobile phone was a worry, and some participants saw their own compulsion (for example to constantly check the display) as alien. High cost was another area of concern. The most commonly mentioned potentially destructive aspect of personal use was dependency or compulsiveness, but economic effects were also a factor. The increased risk of being mean to others with short messages was mentioned. Some participants considered not being available or reachable to be the biggest problem.

Other effects of mobile phone use that could have a bearing on mental health were qualitative changes in communication, for example more, but less personal, contacts; SMS language style; less respect for others' privacy; spontaneity leading to impulsiveness; fragmentation; and risks of misunderstandings and negative messages. Positive aspects included utility, flexibility, and easy access.

### Summary of mobile phone use and mental symptoms

High quantity of use due to demands and expectations for availability at all times was a central area of concern. Demands for availability originated not only from work and the social network, but also from the individual's own ambitions or desires. This resulted in disturbances when busy or resting, the feeling of never being free, and difficulties separating work and private life. Unreturned calls or messages led to overload and feelings of guilt. Personal dependency was an area of concern, as was worry about possible hazards associated with exposure to electromagnetic fields. User competence issues were mentioned, as well as costs. Bad quality of communication was another aspect mentioned, with the mobile phone perceived to increase the risk of receiving or sending negative messages. The major stressor for many, however, was not being available.

### Comparing the results: high computer use versus high mobile phone use

There were several similarities in the factors described as linking computer and mobile phone use, respectively, to mental symptoms. Issues concerning high quantity of use, demands of availability, dependency, disturbed recovery, and mental and communication overload seemed to be common to both technologies. Concerns about the quality of communications were also prevalent in relation to both. Work-related demands for tasks and achievement were more highly related to computer use, while demands for availability regardless of time and space were more related to mobile phone use. Thoughts about increased social isolation were more evident in relation to computer use. Worry about electromagnetic radiation was present in relation to both technologies, but more obvious in relation to mobile phones. User problems or competence issues were prevalent in both areas, although computers seemed to generate more general frustrations.

In our opinion, there were sufficient similarities between the perceived effects of the two technologies to allow the proposal of a combined model of ICT, encompassing computer use, mobile phone use, and their perceived connections to mental symptoms.

### A model of possible paths for associations between ICT and mental symptoms

**Figure 1:**
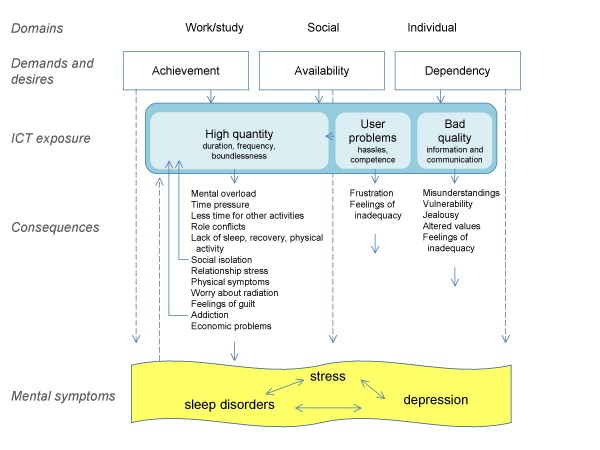
**A model of possible paths for associations between ICT use and mental symptoms**. The proposed model is based on the concepts and ideas expressed by the young adults interviewed in our study.

Our proposed model includes pathways to stress, depression, and sleep disorders, via the consequences of high quantitative ICT use, negative qualitative use, and user problems. The central factors appearing to explain high quantitative ICT use were demands for and expectations of achievement and availability, originating from the domains of work, study, social life, and individual aspirations. These demands could also be direct sources of stress or symptoms of mental ill health. Another central reason for high use was personal dependency. Dependency or compulsion towards ICT could also be a direct source of stress or a correlate to mental ill health. The consequences of high quantitative use included mental overload (interruptions, distractions, multi-tasking, speed of processing), role conflicts, time pressure, less time for other activities, neglecting bodily signals and personal needs (physical activity, nutrition, recovery, sleep), feelings of guilt due to unreturned messages, relationship stress, social isolation, physical symptoms, worry about electromagnetic radiation, addiction, and economic problems. Some factors could be part of a negative loop; high quantitative use could lead to social isolation or addiction, which in turn could generate even heavier use. Destructive communication and information could also have consequences including misunderstandings, vulnerability, effects on attitudes and values, and feelings of inadequacy. User problems including competence issues could be a source of frustration and feelings of inadequacy, and add to the time spent on ICT. In addition to these pathways leading from ICT use to symptoms, there were ideas expressed concerning paths leading in the other direction, for example mental ill health leading to an altered ICT use (higher or lower). Positive effects of ICT use on mental health were also considered to be a possibility, but are not included in the illustrated model. (See Figure 1 below.)

## Discussion

The results and the proposed model are based on the dictums and perceptions of the young adult high ICT users interviewed. The significance of the results and the plausibility of the model should be discussed and verified in relation to state-of-the art knowledge of factors influencing mental health. The participants as a group perceived connections between ICT use and mental symptoms. Some were more prone to identify themselves within the context of these connections, while others maintained that the connections did not apply to themselves. Some respondents thought that factors co-varying with ICT use, such as work- or study-related demands for achievement, or individual characteristics, were the true causes of stress or mental symptoms, regardless of ICT use.

The concept of (extrinsic) demands in our model is represented in well-known psychosocial stress models, for example the models of demand-control [14] and effort-reward imbalance (ERI) [15]. Chronic psychosocial stress, as defined in these models, has an established association with depression [15]. This also applies to young adults; in a prospective study, previously healthy young adults who were exposed to high psychological demands at work had a twofold risk of developing major depressive disorder and generalized anxiety disorder [16]. In our study, demands related to work or study could result in periodic work or study overload with long hours at the computer. Computer tasks such as programming were considered to be especially demanding. The IT work field, with its limitless work hours and tight deadlines, is known to be stressful [17]. Long work hours in general have a broad impact, leading to less time for sleep and recovery, longer exposure to workplace hazards and demands, and less time to attend to private life and family [18]. Other causes of high ICT use in the present study included perceived demands for availability and social interactions. Constant availability via ICT could be a direct link to stress, implying difficulties separating the domains of work and personal life and possibly leading to role conflicts and work/non-work interference. The boundless work situation offered by the use of ICT puts high demands on the individual's own capacity to set limits for work. This could be especially difficult for people with high intrinsic demands in terms of achievement or performance. The concept of demands originating from the individual's own aspirations in our model is comparable to individual characteristics such as overcommitment in the ERI stress model [15] and performance-based self-esteem [19], the latter of which has been suggested as a risk factor for burnout.

The effects of ICT on communication and social relations are vast. ICT not only provides the means to develop, expand, and maintain large social networks via email, SMS, web forums, chat rooms, and so on, it also makes it possible to engage in several lines of communications simultaneously. Chatting or instant messaging seemed to be a central and time consuming activity for many of the computer users in our study. Social support is a factor that promotes health and buffers the negative effects of high psychosocial demands [20,21], and could be considered a positive aspect of ICT use. However, ICT also provides the means of communication overload. Distractions and dual-tasking are demanding on working memory [22,23]; similarly, the participants in our study said that expected immediacy of communications, as evidenced by reminders and demands for explanations of any unavailability, added to mental overload and feelings of guilt. There were also misgivings about the quality of communications and information via ICT, as it was thought to lead to misunderstandings, increased risk of sending or receiving negative messages, and feelings of vulnerability. Studies have shown prospective associations between chatting or instant messaging and perceived stress, depression, and compulsive internet use [6,24]. Furthermore, mobile phone use has been found to distract attention and affect driving and pedestrian safety [25]. Frequently-ringing mobile phones have been shown to enhance allergic responses in patients with atopic eczema or dermatitis syndrome [26], implying that the exposure related to availability is stressful. Another view of this sees social isolation as a possible consequence of high computer use. Some of the participants in our study who spent long hours at the computer said that they had difficulties developing and maintaining social relations outside of the internet. They longed for more real-life friends, but felt more secure at the computer. Whether loneliness is an effect of high computer use or computer use an effect of loneliness is an interesting topic for discussion. Morahan-Martin et al [27] found support for the position that loneliness leads to increased internet use; lonely individuals were more likely than their non-lonely counterparts to use the internet for social interaction and emotional support. Lonely individuals also reported that their internet use interfered with real-life social activities, which could imply a negative loop. Caplan [28] concluded that social anxiety rather than loneliness explains the preference for online social interaction. For socially anxious people, online communication could have a number of benefits in comparison with face-to-face communication, including having control of self-presentation, phrasing, and the speed of the interaction, and thereby feeling safer and more confident than during interactions in person. In a recent prospective study among adolescents, loneliness was negatively related to instant messaging six months later [24], indicating that those with a high level of loneliness were not more drawn to this type of communication. It seems that the connection between ICT use and the social network is complex; however, the impacts, both positive and negative, should be of interest in our model.

Constructs such as internet addiction, problematic internet use, and compulsive internet use have been previously discussed [29], as has the term "problem mobile phone use" [30]. Internet addiction has even been proposed as a specific psychiatric illness [31], though another view holds that problematic internet use shares elements with impulse control disorders and is related to specific activities like gambling or accessing pornography, not to the internet per se [10,29]. In our study, some respondents claimed a compulsion towards the mobile phone or computer, feeling an urgent need to check for messages or other information. Game playing was a central activity, and some participants who spent a lot of time playing computer games (online or offline) admitted to neglecting other activities such as social life, physical activity, or sleep.

Another question raised in our study was that of possible negative effects, such as increased aggression or altered perceptions of reality, due to violent content in games. Grusser et al [32] found only weak evidence for the assumption that aggression is related to excessive gaming, but concluded that there is addictive potential in gaming. An evaluation of the short-term physiological and psychological effects of playing violent videogames, compared to non-violent games, revealed effects on arterial pressure and state of anxiety, but not on measures of hostility [33]. A study among mental health professionals in the USA revealed that when clients presented problematic internet use as a primary problem, this most likely included use of pornography [34]. Another study [35] found erotica to be the best prospective predictor of compulsive internet use. In contrast, use of pornography was barely mentioned in our study. We did not ask any direct questions aimed at the subject, but the interviewer did ask questions about possibly destructive personal uses. Hence, either the use of pornography was not perceived by the participants as a problem, or it was a subject they did not wish to discuss.

Online gambling is another possible hazard, though most of those in our study who gambled considered themselves to be in control of the problem. Increased online gambling addiction has been noted in Sweden. It is likely that easy access contributes to the increase; gambling can take place in the privacy of the home, just "one click away". Another growing problem in Sweden is young people in debt because of applying for and accepting bank loans or credit via SMS, or because of high phone bills. Dependency, compulsion, or addiction, whether it concerns chatting, game playing, gambling, or other aspects of ICT use should be of relevance in our model. Addiction could be a link to mental ill-health, regardless of ICT use. It could also be argued that ICT in itself is potentially addictive. Some participants in the study claimed that they felt compulsive regarding their mobile phone, but not in any other areas of life.

User problems, including technical frustrations and competence problems, are associated with stress [36] and were a cause of irritation in our study, adding to workload and causing more time to be spent at the computer than planned. Users' great dependency on ICT for work, leisure, information, and communications makes user problems especially disturbing. Bad ergonomics and physical symptoms associated with ICT use were considered risk factors for mental health issues in this study. Computer use and mobile phone use have been associated with musculoskeletal complaints [37,38], which in turn can have a negative effect on mental health. Furthermore, the sedentary nature of computer use and the neglect of physical activity were common complaints in our study. A high level of sedentary leisure activities negatively affects mental health [39], while physical activity has positive effects on mental health and is acknowledged as a possible complementary treatment for depression and stress-related disorders [39,40]. The impact of ICT use on physical symptoms or physical activity should be of interest in our model. Worrying about possible exposure to electromagnetic radiation as a consequence of high ICT use was another source of stress in our study. Some participants even proposed radiation as a possible cause of mental ill-health. Exposure to electromagnetic fields due to ICT use is currently not known to have any major health effects [41], though there is some inconsistency in the published results [42]. A study of skin complaints associated with computer work suggested that there were factors other than electromagnetic radiation contributing to the symptoms [7]. It was hypothesized that occupational strain resulted in psychophysiological stress reactions (termed *techno-stress*) that were conditioned to the computer work environment, and that this was why symptoms appeared or worsened in connection with the computer. Perceived electrosensitivity is associated with reporting symptoms of depression and worse general health than controls [43]. Worrying about exposure to electromagnetic radiation is probably of greater significance for mental health than actual exposure, and could be of interest in our model.

Prevention of mental disorders such as depression is of course of great importance. Psychosocial function in young adults is affected by depression [44], and cognitive impairments are common [45]. Gender, socio-demographic factors, general health, and major life events, as well as individual factors such as coping skills, are all related to the incidence of depression [46-48] among young people. Whether computer use or internet use can lead to depression has been the subject of debate; studies have shown mixed results [8,49-51]. Bell [9] points out that the internet is just a medium, and cannot in itself be considered good or bad for mental health. Mental health information can be found on the internet, as well as online support groups, online therapy, and more. For a review of internet-based mental health interventions, see Ybarra and Eaton [52]. On the other hand, the internet also provides information and advice on suicide methods, enables interaction with other people contemplating suicide, and can provide the means to suicide via online shopping [53]. The internet can also be a medium for bullying via email and web pages, as can mobile phones via text messages and the misuse of picture-taking abilities [54]. Internet or mobile phone bullying can be done anonymously and can affect victims in the privacy of their own homes. Almost one fifth of Swedish adolescents (12-16 years) had experienced being bullied via the internet [55]. Hence, negative content is another major factor in ICT use, with potential effects on depression and other mental symptoms.

Too little sleep, dislocated sleep, or difficulties falling asleep because of sitting up late at the computer were prevalent in our study, along with sleep disturbances caused by late night phone calls or SMS messages. Intensive ICT use which negatively affects sleeping habits has been associated with poor perceived health among adolescents [56]. In a study of Korean university students, 30% reported insufficient sleep and of these about a third pointed to visual media including computers as the primary reason [57]. Other factors associated with insomnia and depression among young adults are negative family life stress and academic stress [58]. The importance of sleep and recovery for physical as well as mental health is well known. Insomnia affects psychosocial functioning and increases subsequent risks for somatic health problems, interpersonal problems, and psychological problems in adolescents [59]. Insufficient sleep has been shown to be a risk factor for reporting poor self-rated health among young adults [60]. Furthermore, sleep disorders are a predictor of major depression onset, with an odds ratio of nearly 4, according to a prospective study of young adults [61]. The negative impact of ICT use on sleep should be of major concern in our model.

### Limitations of the study

This interview study was performed with a highly selected study group so as to enhance the potential to identify factors or conditions that could connect ICT use with mental symptoms. There is a possibility that the current mental states of the subjects affected their perceptions. We had an even gender distribution in the group as a whole, but the high mobile phone users included more women than men. We have not pursued the issue of gender differences, though they certainly could exist. There are gender differences in ICT usage [62], as well as in prevalence of mental symptoms [1]. The study group was also highly selected in that it consisted of students from high achieving academic programs. Neither socio-economic factors nor academic background were taken into account in the present study. Another limitation is that the participants sometimes spoke in more general terms, rather than through personal experience, which means that some of the factors that emerged could be mere speculation. On the other hand, we were primarily interested in concepts and ideas. We have not challenged the participants' thoughts about stress, depression, and sleep disturbances.

### Implications for future studies

Most of the factors and conditions in the model can be argued to influence mental symptoms. Some factors are probably more central; for example, work load, psychosocial demands, addiction, impacts on sleep, and social support, as well as individual characteristics or vulnerabilities. Different exposure profiles might co-occur with different individual characteristics and lifestyle factors, leading to a variety of possible pathways. One person could be active and socially outgoing, carry a high workload and other demands, use computers and mobile phones heavily in both work and social life, and lead a lifestyle with insufficient recovery. Another person might spend much of the day and night at the computer, playing interactive games online, carrying on a social life on the internet but having few friends "irl", experiencing dislocated sleep, and leading a sedentary lifestyle. Methods of testing the model could include combining different ICT exposure factors (high/low), adding psychosocial demands, dependency and lifestyle factors (sleep, physical activity, social support), and using mental symptoms as outcomes in an epidemiological, preferably longitudinal, study. It seems likely that tailoring is necessary when designing interventions and intervention studies.

## Conclusions

The concepts and ideas expressed by the young adults reporting high ICT use and mental symptoms generated a model of possible paths for associations between ICT exposure and mental symptoms. Demands for achievement and availability as well as personal dependency were major causes of high ICT exposure, but were also direct sources of stress and mental symptoms. The proposed model shows that factors in different domains may have an impact and should be considered in epidemiological and intervention studies.

## Competing interests

The authors declare that they have no competing interests.

## Authors' contributions

ST and MH conceived of and designed the study. ST performed the data collection and data analysis, and drafted the manuscript. LD and AH participated in interpreting data and developing the model. LD, AH, and MH helped to draft the manuscript. All authors have read and approved the final manuscript.

## Pre-publication history

The pre-publication history for this paper can be accessed here:

http://www.biomedcentral.com/1471-2458/10/66/prepub
